# Somatic Mutations in KEAP1-NRF2 Complex in Breast Cancer

**DOI:** 10.3390/cancers16132411

**Published:** 2024-06-29

**Authors:** Micaela Almeida, Catarina L. Ferreira, Rosa Maria Tomé, José Fonseca-Moutinho, António Polónia, Ana Cristina Ramalhinho, Luiza Breitenfeld

**Affiliations:** 1 Health Sciences Research Centre (CICS), Faculty of Health Sciences, University of Beira Interior (UBI), Avenida Infante D. Henrique, 6200-506 Covilhã, Portugal; cferreira@fcsaude.ubi.pt (C.L.F.); jafmoutinho@fcsaude.ubi.pt (J.F.-M.); luiza@ubi.pt (L.B.); 2 Clinical Academic Centre of Beiras (CACB), Edifício UBImedical, Estrada Municipal 506, 6200 Covilhã, Portugal; rosatome@chcbeira.min-saude.pt; 3 Department of Pathology, Ipatimup Diagnostics, Rua Júlio Amaral de Carvalho, 45, 4200-135 Porto, Portugal; apolonia@ipatimup.pt; 4 Cova da Beira Local Health Unit, Alameda Pêro da Covilhã, 6200-251 Covilhã, Portugal; 5 Escola de Medicina e Ciências Biomédicas, Instituto de Investigação, Inovação e Desenvolvimento, Fundação Fernando Pessoa (FP-I3ID), Avenida Fernando Pessoa, 150, 4420-096 Gondomar, Portugal

**Keywords:** breast cancer, NRF2, KEAP1

## Abstract

**Simple Summary:**

Breast cancer remains a burden for women worldwide. Among the factors that contribute to breast cancer development is estrogen exposure during one’s lifetime, both endocrine and exocrine. Polymorphisms in KEAP1-NRF2 complex, the master regulator of oxidative stress metabolism, have been correlated with a poorer outcome. The aim of our study was to identify and correlate polymorphisms in this complex, in breast cancer tissue, benign surrounding tissue and in peripheral blood. We verified a tendency towards the loss of heterozygosity in the benign surrounding tissue when compared to blood and to tumour tissue. Correlating with clinical data, histologic grade 2 has a higher variability of genotypes. The results indicate a heterogeneous and active microenvironment. Therefore, clinical approaches would benefit from the evaluation of somatic mutations in this complex. Moreover, further studies should be developed in order to evaluate the predictive value of the histologic grades, taking into consideration the genetic profile.

**Abstract:**

Breast cancer remains the leading cause of cancer deaths for women. Long-term estrogen exposure is considered carcinogenic due to semiquinone production and to compromised detoxification. Metabolic regulator polymorphisms, such as *KEAP1* (rs1048290) and *NRF2* (rs35652124, rs6721961, rs6706649), can be valuable in understanding the individual cytoprotection profile. Thus, we aim to genotype these polymorphisms in blood, tumours and surrounding tissue, to identify somatic mutations and correlate it to prognoses. A total of 23 controls and 69 women with histological confirmed breast cancer were recruited, and DNA from blood/surrounding/tumour tissue was genotyped. Genotyping and clinicopathological data were correlated. We verified that rs35652124 presents different genotype distribution between the blood/surrounding tissue (*p*-value = 0.023) and tumour/surrounding tissues (*p*-value = 0.041). Apart from rs35652124 and considering the histological grade, the other four polymorphisms have different distributions among different tissues. There is a tendency towards the loss of heterozygosity in the surrounding tissue when compared to blood and tumour tissues, and higher genotype variability in histologic grade 2. These somatic mutations and different distribution patterns may indicate a heterogeneous and active microenvironment, influencing breast cancer outcome. Additionally, it would be pertinent to evaluate the predictive value of the histologic grade 2 considering somatic mutation profiles and distributions.

## 1. Introduction

Breast cancer remains the type of cancer with the highest incident rate in women worldwide and the leading cause of cancer death in women in 123 countries [1]. The development of breast cancer is due to several factors, among them endogenous and exogenous hormones exposure, compromised immunological system, metabolic imbalance, oxidative stress and genetic alterations [2,3].

Estrogen exposure is widely described as a carcinogenic factor for breast cancer development, which may be due to endocrine or exocrine estrogens [4]. Genomic instability and somatic mutation acquisition might allow for the carcinogenic effect of estrogen, whether through increased estrogen levels or through inefficient estrogens detoxification due to a compromised metabolism [5,6].

The single nucleotide polymorphisms (SNPs) in the metabolic pathway of estrogen might compromise the induction of phase II detoxifying enzymes, responsible for the transformation of endogenous and exogenous compounds into a more excretable form [7]. Low levels of phase II enzymes result in an estrogen increase, contributinsg to quinone accumulation and eventually leading to carcinogenesis [7]. Previous studies performed by our research team indicate that the metabolic imbalances due nuclear factor Erythroid 2-related Factor 2 (NRF2) and Kelch-Like ECH Associated Protein 1 (KEAP1) lead to poor outcomes in breast cancer patients [8,9].

The transcription factor NRF2 is encoded by the *NF2EL2* gene, commonly known as the *NRF2* gene, located at chromosome 2 (2q31.2) [10]. The negative regulator of NRF2 is KEAP1 encoded by the *KEAP1* gene, located at chromosome 19 (19p13.2) [11]. The complex KEAP1-NRF2 is a major regulator of phase II metabolism. In the presence of radicals produced by internal or external aggressions, the NRF2 that normally binds to KEAP1 in the cytoplasm translocates to the nucleus and binds to antioxidant responsive elements (AREs), small Maf proteins and DNA, leading to the release of phase II enzymes [12]. The reactive oxygen species are then eliminated and a basal state is achieved. KEAP1 acts as a negative regulator of NRF2. Once it enters the nucleus it binds NRF2 and takes it back to the cytoplasm to suffer proteasome degradation [13].

However, SNPs in *NRF2* and/or *KEAP1* might interfere with the function of the complex. The mutated allele of rs1048290 (C > G) polymorphism in *KEAP1* increases KEAP1 expression and increases the capability of KEAP1 to bind NRF2, leading to decreased levels of NRF2 in the nucleus, with a consequent decrease in detoxification enzymes and to a metabolic imbalance due to a compromised metabolism [9,14,15]. The rs35652124 (A > G), rs6721961 (C > A) and rs6706649 (G > A) polymorphisms of *NRF2* also exert a similar effect on the metabolism. These polymorphisms are related to a lower expression of NRF2 levels, leading to a decrease in the expression of phase II enzymes and consequently compromising cytoprotection [9,16]. The effects of these polymorphisms in genomic DNA extracted from peripheral blood have been described in several pathologies [17,18]. However, on what concerns breast cancer, fewer studies have been performed. Hartikainen et al., in 2015, associated the carriers of the altered allele of rs1048290 with a higher KEAP1 expression and shorter relapse-free survival [14]. Relative to *NRF2*-selected polymorphisms, women with a homozygous (AA) genotype of rs6721961 had an increased risk of breast cancer development [16]. These polymorphisms are related to a higher expression of KEAP1, increasing the binding of KEAP1 to NRF2, and to low levels of NRF2. Both polymorphisms, studied by Hartikainen and colleagues, might be related to breast cancer development once cytoprotection is compromised due to low levels of NRF2 in the nucleus, leading to lower induction of phase II enzymes [14,16].

Nevertheless, there is a lack of studies evaluating the presence/absence of the altered allele of these polymorphisms in tumour tissue and in the surrounding tissue. In this regard, we aim to evaluate and compare the genotype of these SNPs of *KEAP1* (rs1048290) and of *NRF2* (rs35652124, rs6721961, rs6706649) in blood, surrounding tissue and tumour tissue, and correlate it with clinicopathological data to evaluate a possible relation with breast cancer prognosis.

## 2. Materials and Methods

### 2.1. Study Population

The participants recruited for the study were women followed by the Child and Women Health Department, Gynaecology Oncology Division of Cova da Beira Local Health Unit, Covilhã, Portugal, who agreed to take part in the study and signed the informed consent form. The recruitment period was from December of 2016 until June of 2020. The study was approved by the Institutional Review Board of Cova da Beira Local Health Unit with the numbers n.° 28/2008 and an addendum in 17 August 2016; further approval for new data acquisition, n.° 20/2023.

A total of 92 women were recruited. The inclusion criteria for the control group were women 18 years old or older, with no previous history of breast cancer and without family history of cancers. The exclusion criteria were women with amenorrhea, previous history of hysterectomy or oophorectomy.

For the patient group, 69 women were recruited. The inclusion criteria were women 18 years old or older that underwent fine needle biopsy and had a breast cancer diagnosis. The exclusion criteria were women with amenorrhea, previous history of hysterectomy or oophorectomy. After the tumour was surgically removed and analysed by pathology, it was found that six cases were not hormone-dependent breast cancers (ER^+^) and were therefore excluded from the study. Thus, the total number of breast cancer patients included in the study for further analysis was 63.

### 2.2. Sample Collection

The samples of the patients consisted of peripheral whole blood and slides from the tumour and from the surrounding tissue of the breast cancer patients.

The peripheral whole blood was collected by venous puncture to further DNA extraction.

Through hematoxyline–eosine slides, the medical pathologist of the research team identified the blocks with tumour tissue and with surrounding tissue. For each patient there were selected two blocks, one with tumour tissue and another one with surrounding tissue.

At the Anatomic Pathology Service of Local Health Unit of Cova da Beira, 10 μm slides from each block were cut and assembled.

### 2.3. DNA Extraction from Peripheral Blood

The Wizard Genomic DNA purification kit (Promega) was used to isolate DNA from peripheral blood collected on EDTA tubes. The manufacturer’s instructions were followed, and the DNA extraction sample was stored at 4 °C.

### 2.4. DNA Extraction from Paraffin-Embeded Slides

Prior to DNA extraction, hematoxylin and eosin (H&E) staining was performed in order to evaluate the integrity of the morphology. H&E staining allowed us to carry out a morphological control, verifying the integrity of the morphology and the presence of tumour tissue and of benign surrounding tissue. For DNA extraction from paraffin-embedded slides, an extraction protocol was optimized. Firstly, in order to deparaffinizate, the slides were fully submerged in xylene on a 50 mL falcon. Incubation was performed at room temperature for 7 min. After, the slides were submerged in absolute ethanol for 5 min at room temperature. The slides were left to air-dry for 5 to 10 min. All the material was carefully manipulated in order to avoid cross-contamination. The tissue was then scraped from the slide.

The tissue from each slide was scraped off with a new scalpel and placed in previously identified 1.5 mL microtubes. After paraffin removal, the samples were ready for genomic DNA extraction, which was performed using the QIAmp DNA FFPE Tissue Kit (Qiagen, Germantown, MD, USA) according to the manufacturer’s instructions). The DNA extraction from paraffin-embedded slides was performed on an extraction cabinet and filtered tips were used to avoid contamination.

Following the protocol, the DNA samples were stored at 4 °C once the real-time PCR was performed, since the real-time PCR was carried out within the time period in which the samples could be stored at 4 °C. They were subsequently stored at −20 °C.

### 2.5. NRF2 and KEAP1 Sequencing

The amplification of the regions of interest of *NRF2* and of *KEAP1* from genomic DNA extracted from peripheral blood were amplified by conventional PCR as described by Almeida, M. et al., 2019 [9].

The amplification of the fragments of *NRF2* and *KEAP1* were performed using Real-Time PCR and each master Mix had a total volume of 20 μL, containing 0.3 μM of each primer, 10 μL of Maxima SYBR Green qPCR Master Mix (Thermo Scientific, Waltham, MA, USA) and 100 ng of genomic DNA.

For the polymorphisms rs35652124, rs6721961 and rs6706649 of *NRF2* the primer set was the same one used for conventional PCR. The Real-Time PCR conditions were as follows: initial incubation at 95 °C for 10 min, followed by 70 cycles at 95 °C for 10 s, 60 °C for 10 s and at 72 °C for 20 s, finally the temperature of the reaction mixture was increased up to 95 °C at a rate of 0.1 °C/s, starting at 68 °C for 15 s.

For *Keap1* rs1048290 polymorphism the primer sets were designed with Primer3Plus:

Forward primer: 5′-TTGCAGGTATGAGCCAGAGC-3′;
Reverse primer: 5′-GATGGTAGGGGGTGTTCCTG-3′.

The Real-Time PCR conditions were 95 °C for 10 min, 70 cycles at 95 °C for 10 s, 57 °C for 10 s and at 72 °C for 20 s. The reaction mixture temperature was increased up to 95 °C at a rate of 0.1 °C/s, starting at 68 °C for 15 s.

The amplified fragments of *NRF2* and *KEAP1* were sequenced through Sanger sequencing (performed by STABVIDA), and the sequencing results were analysed in ChromasPro version 2.1. Three researchers evaluated the sequencing results independently and 10% of the samples was randomly selected and re-evaluated. All results were in accordance.

### 2.6. Statistical Analysis

The statistical analysis was performed using IBM SPSS statistics version 25. A Chi-squared test was performed, considering a statistical significance when *p*-value was <0.05.

## 3. Results

For the present study, a total of 23 controls and 63 breast cancer patients were included. The characteristics of the study population are described in Table 1.

All genomic DNA, extracted from peripheral blood, was sequenced for rs35652124, rs6706649 and rs6721961 polymorphisms of *NRF2* and for rs1048290 of *KEAP1*.

The Hardy–Weinberg equilibrium was calculated for the control and patient genotypes. Breast cancer risk was also calculated (Table 2).

The Hardy–Weinberg equilibrium is similar for the populations in the study (controls and cases, *p*-value ≥ 0.05). In terms of assessing the risk for the carrier allele, it was found that none of the genotypes under study had an increased risk value for developing breast cancer (*p*-value ≥ 0.05).

In addition to peripheral blood, we obtained samples of tumour surrounding tissue and tumour tissue for each woman. Sequencing was performed for each (blood, surrounding and tumour tissues), although we were not able to obtain DNA from all tissues of each sample. For DNA extracted from paraffin-embedded slides the success rate was not the same. For *NRF2*: 26 (92.9%), samples of surrounding tissue were successfully amplified and 22 (78.6%) samples of tumour tissue were amplified (Table 3). The *KEAP1* fragment was successfully amplified in 23 (82.1%) and in 19 (67.9%) surrounding and tumour tissue samples, respectively. Therefore, from a total of 28 breast cancer cases, 16 were successfully genotyped for the three types of tissue (blood, surrounding and tumour tissue) for *NRF2* SNPs (rs35652124, rs6706649 and rs6721961) and for rs1048290 of *KEAP1*.

The study of the allele and genotype frequencies lead us to include only cases that have been simultaneously amplified in blood, surrounding tissue and tumour tissue for this analysis. Therefore, 21 cases were successfully amplified, for all tissues, for rs35632124, rs6706649 and rs6721961 of *NRF2*. The Hardy–Weinberg equilibrium was calculated for each polymorphism for each population of tissues, taking into account the genotype frequencies (Table 4) and allele frequency (Table 5).

As can be seen in Table 3, there is a significant difference (*p*-value < 0.001) concerning rs6706649. This polymorphism has a different frequency distribution in the surrounding tissue (Hardy–Weinberg equilibrium is not observed).

Table 4 allows us to compare the allele frequency of the three tissues studied (blood, surrounding tissue and tumour tissue).

As we might observe, the only significant difference observed is related to the rs6706649 of *NRF2* (*p*-value = 0.007). There is a great number of A allele carriers (mutated allele) when compared with the wild-type allele (G).

When the distribution of the alleles is compared with tumour tissue no significant differences are verified.

The genotypes frequencies were correlated for the different tissues in order to verify if the genotype distribution is the same between the different types of tissue.

The genotypes frequency between the surrounding tissue and the blood is significantly different for rs35652124 (*p*-value = 0.023) (Table 5). There is a decrease in the heterozygous genotype frequency from blood (42.9%) to the surrounding tissue (19%). There is also a significant difference between the genotype frequency of the tumour and of the surrounding tissues (*p*-value = 0.041). There is a decrease in the homozygous genotype frequency and an increase in the heterozygous genotype in tumour tissue, which we can also observe in blood tissue.

For the remaining polymorphisms under study, no statistical significance was found. However, there seems to be a trend: the surrounding tissue has a different genotypes distribution when compared to other tissues (blood and tumour).

Based on the above results, it was considered pertinent to study the relationship between the distributions of the different genotypes in the tissues with clinicopathological factors, such as histologic grade (Table 6).

With the exception of the rs35652124 polymorphism, the genotype distribution for each histologic grade is different among the tissues.

**Figure 1:**
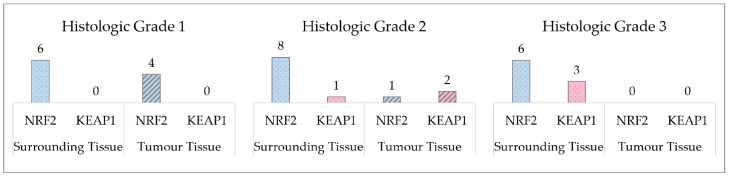
Number of acquired mutations in the surrounding and tumour tissues, when compared to the genotype profile of the blood.

In this particular study, only the cases that were genotyped for the three tissue types were analysed. However, due to the limited size of the sample, it was considered pertinent to analyse the variation in genotypes for each polymorphism, in each histological grade (Figure 1).

In Figure 1 we can observe the count of genotypes in the tissues that are different when compared to blood genotypes. In histologic grade 1 the number of genotypes that are different from the blood genotypes is higher in the surrounding tissue. In histologic grade 2 we can observe a greater difference in the *NRF2* polymorphism count, as in histologic grade 1. In addition, there are genotypes differences in *KEAP1*. In histologic grade 3 there is no difference in tumour tissue genotypes, however, surrounding tissue has a greater number of genotypes.

## 4. Discussion

Metabolic imbalance has long been identified as one of the factors for poor prognosis, increased recurrence, decreased disease-free survival and resistance to therapy. Among the factors that may play a leading role in breast cancer metabolism is the KEAP1-NRF2 complex, which regulates the metabolism and, therefore, the response to oxidative stress. The *NRF2* rs35652124, rs6706649 and rs6721961 polymorphisms are related to a decrease in NRF2 transcription, which may compromise the response to oxidative stress. The *KEAP1* rs1048290 minor allele has also been associated with impaired response to oxidative stress, since it is related to higher KEAP1 protein expression and with an increased association between KEAP1 and NRF2, with the NRF2 becoming less available to enter the nucleus and promote the transcription of phase II enzymes [14,15].

It is therefore considered pertinent to evaluate the genotypes of these polymorphisms in the tumour and in the benign tissue surrounding the tumour. Starting from the genotype of the peripheral blood, the evaluation of somatic mutations in tumour tissue and in the benign tissue that surrounds the tumour might be pertinent to better understand if they have a pathological effect in breast cancer development and aggressiveness [19].

In the present study women with diagnosed breast cancer (*n* = 63) and women with no history of breast cancer (*n* = 23) were included. Genomic DNA from peripheral blood was sequenced for rs35652124, rs6706649, rs6721961 of *NRF2* and rs1048290 of *KEAP1*. The Hardy–Weinberg equilibrium was evaluated and it was not verified as deviating from the HWE (*p*-value > 0.05) for both controls and patients, as expected for low-penetrance genes. After, the risk for developing breast cancer was assessed and an increased risk for breast cancer development was not found in the population under study (*p*-value > 0.05). These results are not in accordance with the ones verified by Hartikainen et al. for rs6721961 and for rs1048290 [14,16]. This is possibly due to the effect of a lower sample size. Nevertheless, there is a lack of studies correlating all these polymorphisms of the metabolic pathway with the risk of breast cancer development. Similarly, there is a lack of studies assessing the genotypes of the referred polymorphisms and tumour tissues, as described in the present study. Genomic DNA was extracted from peripheral blood from 56 paraffin-embedded tissue slides (28 slides of surrounding benign tissue and 28 slides of tumour tissue). Most likely, the lack of studies using these source materials is due to the difficulty in extracting genomic DNA from paraffin-embedded tissues. The long-term storage and the possibility of DNA degradation might compromise the results. Moreover, the quality and quantity of the genomic DNA extracted might not be ideal. In the present study, the DNA was extracted from 10 μm slides and instead of a conventional PCR, a real-time PCR with a high number of cycles was performed (70 amplification cycles) in order to guarantee enough template DNA for Sanger sequencing. Even so, not all samples were amplified and genotyped. For the polymorphisms of *NRF2*, a total of 21 cases were properly sequenced for the tree types of tissue (blood, surrounding benign tissue and tumour tissue). For *KEAP1*, a total of 16 cases were sequenced for the three types of tissue. This is a limitation of the study, however, due to the source of the material it is a successful rate.

This step led to the study of the HWE for all tissues and for each polymorphism. The principle of the HWE was verified for the majority of the polymorphisms, with the exception of the rs6706649 in the surrounding benign tissue, which was in linkage disequilibrium (LD) (*p*-value < 0.001). Assessing the allele frequencies, it was verified that the mutated allele (A) frequency is higher in the surrounding benign tissue when compared to blood, suggesting somatic mutations in the surrounding benign tissue.

Therefore, a correlation between the genotype distribution of rs35652124, rs3706649, rs6721961 of *NRF2* and rs1048290 of *KEAP1* in the three different types of tissues (blood, surrounding benign and tumour tissues) was performed. For rs35652124, a significant difference in the genotype distribution was found between blood and surrounding benign tissue (*p*-value = 0.023) and between tumour tissue and surrounding benign tissue (*p*-value = 0.041). The surrounding benign tissue had somatic mutations that reflect a tendency towards a lower heterozygous genotype (nine cases in the blood and four cases in the surrounding tissue) and increased homozygous genotypes (wild-type and mutant). Although this difference in the profile of the surrounding benign tissue is more prevalent in rs35652124, all the polymorphisms studied show genotypic differences between the tissues, highlighting the loss of heterozygosity in the surrounding tissue. The loss of heterozygosity in benign breast tissue was associated with an increased risk for breast cancer development by Euhus et al., 2002 [20].

The increase in somatic mutations in benign tissue has long been correlated with age and consequently with cancer development [21,22,23]. Thus, a correlation between genotype alterations among the tissues and the histologic grade of the breast cancer was performed. It was verified that, in addition to the tendency already referred to for the loss of heterozygosity, the histologic grade 2 tends to have a different genetic profile from histologic grade 1 and 3.

These results lead to several questions. Why are there no variations in *KEAP1* polymorphism in histologic grade 1 cases? Why is the variability in genotype profiles greater in surrounding benign and tumour tissues in breast cancer with histologic grade 2? Why were there no verified alterations in the tumour tissue, when compared to the surrounding benign tissue genotypes, for the four polymorphisms in histologic grade 3 cases?

NRF2 is responsible for the regulation of several cytoprotective genes, like phase II enzymes. The altered alleles of the *NRF2* polymorphisms in this study are related to a lower activity of NRF2 and *KEAP1* rs1048290 is related to an increased affinity to bind NRF2, leading to its lower availability in the nucleus. A possible answer to the first question is that although the transcription of NRF2 is lower or normal, it is still available in the nucleus to trigger the metabolic response. Thus, this protects cells and eventually contributing to a less aggressive development.

The majority of cases had a histological grade 2 and, in this histological grade, we verified a greater variability in genotypes, both in surrounding benign and in tumour tissues. The histological classification, through Nottingham histological grade, results from the evaluation of tubular formation, nuclear pleomorphism and mitosis [24]. Therefore, the Nottingham scale is a prognostic factor that aids clinicians to identify the best approach for each patient. A histological grade 2 is consider a moderate grade [24,25]. However, this classification does not take into account the characteristics of the benign surrounding tissue.

Field cancerization is an assumption that the cells of the surrounding benign tumour tissue have molecular alterations that might not alter the morphology of the tissue and that can develop into malignant cells [26]. Somatic mutations in this surrounding tissue can be acquired throughout one’s life or result from the microenvironment and, in either case, somatic mutations can lead to the development of cancer or they can be alterations that do not trigger cancer [27,28].

Thus, histologic grade 2 breast cancer tumours might have an increased instability than observed, once field cancerization occurs without morphological alterations [29]. The polymorphisms in *NRF2* and *KEAP1* might be indicative of a more heterogeneous and active microenvironment that might confer a survival advantage to cells with compromised cytoprotection, also, higher recurrences might be related to the changes in the surrounding tissue [28,30].

The histologic grade 3 is the grade with a lower prognosis. Considering that cancerization can confer survival advantages to altered cells, it is possible that histologic grade 3 breast cancers are a well-established population of cells that suffered a cancerization process, and on the other hand the surrounding benign tissue is suffering the cancerization process and therefore contributing to a future cancer progression [29].

These findings are of great concern once they could affect the therapeutic approach. In a meta-analysis, our research team verified that breast cancer patients that overexpressed NRF2 had a lower survival compared to the ones that expressed lower levels of NRF2 [8]. The dual role of the complex NRF2-KEAP1 is of main importance. On what concerns breast cancer development, lower levels of NRF2 due to polymorphisms in *NRF2* or *KEAP1* alone or in association with polymorphisms in estrogen biosynthesis and metabolism might contribute to inefficient estrogen detoxification. After the disease develops, high levels of NRF2 will protect cancer cells from oxidative stress and eventually promote resistance to therapeutic approaches. Low levels of NRF2 are related to a better response to therapeutic approaches; however, healthy cells present a low response to oxidative stress, being at risk. This study, in the future, might have an impact on the therapeutic approach once loss of heterozygosity in the surrounding tissue, leading to lower levels of NRF2, may be a worse prognosis. In opposition, a well-stablished population of tumour cells whose genotype is related to increased levels of NRF2 might resist therapeutic approaches.

Our findings are of main interest in increasing the knowledge about somatic mutations in *KEAP1* and *NRF2* in breast tissue (benign and malignant), however, the study was performed in a small sample population. Further studies should be performed on a larger population and follow-up should be performed in order to understand the impact of these mutations in breast cancer overall survival, disease-free survival, relapse-free survival and therapeutic responses. Also, reproductive data should be evaluated such as age at menarche, menopause, full-term pregnancies, abortions, time of breast feeding and hormone replacement therapy.

## 5. Conclusions

Cancerization processes have been debated over the years, and the present study provides evidence that the surrounding tissue might demonstrate more instability, having a genotype profile different from that of tumour tissue.

Similarly, the predictive value of the histologic grade 2 should be re-evaluated considering the metabolic imbalance and somatic mutation profiles.

Overall, the KEAP1-NRF2 complex, in addition to the possible dual role it plays in breast cancer, can also be an important factor for clinical evaluation and surgical approaches.

To our knowledge, there are no similar studies published. It is urgent to replicate this study in a larger population in order to contribute to the mitigation of breast cancer incidence and mortality. This is pertinent, since NRF2 has been associated with a worse prognosis and therapeutic resistance, and limiting genetic evaluation to peripheral blood does not provide a real insight into the acquisition of somatic mutations in tissues [8]. In the future, a more in-depth genetic evaluation could help clinicians achieve a better prognosis for breast cancer patients.

## Figures and Tables

**Table 1:** Characteristics of the studied population.

Parameter	*n* (%)
Controls	
Total	23 (100)
Age	
Mean	52.70
Minimum	24
Maximum	87
Breast Cancer Patients	
Total	63 (100)
Age	
Mean	65.08
Minimum	36
Maximum	95
ER status	
Positive	63 (100)
Negative	0
PR status	
Positive	48 (76.2)
Negative	14 (22.2)
Missing	1 (1.6)
HER2 status	
Positive	8 (12.7)
Negative	54 (85.7)
Missing	1 (1.6)
Histological Grade	
1	12 (19)
2	36 (57.1)
3	15 (23.8)

**Table 2:** Hardy–Weinberg equilibrium (HWE) and breast cancer risk for the polymorphisms under study in blood samples.

SNP	Controls *n* (%)	HWE	Cases *n* (%)	HWE	OR (95% CI) ^1^	*p*-Value
	23 (100)		63 (100)			
*NRF2*						
rs35652124						
AA (wt)	13 (56.52)	0.895	33 (52.38)	0.932	Ref.	
AG	8 (34.78)		26 (41.27)		1.280 (0.462–3.550)	0.634
GG	2 (8.70)		4 (6.35)		0.788 (0.128–4.837)	0.796
rs6706649						
GG (wt)	19 (82.61)	0.293	50 (79.37)	0.961	Ref.	
GA	3 (13.04)		12 (19.05)		1.520 (0.386–5.988)	0.547
AA	1 (4.35)		1 (1.59)		0.380 (0.023–6.386)	0.486
rs6721961						
CC (wt)	18 (78.26)	0.843	48 (76.19)	1	Ref.	
CA	5 (21.74)		14 (22.22)		1.050 (0.331–3.336)	0.934
AA	0		1 (1.59)		NA	0.541
*KEAP1*						
rs1048290						
CC (wt)	8 (34.78)	0.901	18 (28.57)	0.417	Ref.	
CG	12 (52.17)		36 (57.14)		1.333 (0.463–3.843)	0.594
GG	3 (13.04)		9 (14.29)		1.333 (0.283–6.279)	0.715

^1^ OR, odds ratio; CI, confidence interval.

**Table 3:** Hardy–Weinberg equilibrium for each genotype in the different tissues, for the polymorphisms in the study of *NRF2* and *KEAP1*.

SNP	Blood *n* (%)	HWE	Surrounding Tissue *n* (%)	HWE	Tumour Tissue *n* (%*)*	HWE
	21 (100)		21 (100)		21 (100)	
*NRF2*						
rs35652124						
AA (wt)	11 (52.39)	0.884	14 (66.67)	0.094	12 (57.14)	0.974
AG	9 (42.85)		4 (19.05)		8 (38.09)	
GG	1 (4.76)		3 (14.29)		1 (4.76)	
rs6706649						
GG (wt)	19 (90.48)	0.974	15 (71.43)	<0.001 *	16 (76.19)	0.595
GA	2 (9.52)		1 (4.76)		4 (19.05)	
AA	0		5 (23.81)		1 (4.76)	
rs6721961						
CC (wt)	17 (80.95)	0.890	19 (90.48)	0.974	18 (85.71)	0.940
CA	4 (19.05)		2 (9.52)		3 (14.29)	
AA	0		0		0	
*KEAP1*	16 (100)		16 (100)		16 (100)	
rs1048290						
CC (wt)	5 (31.25)	0.802	7 (43.75)	0.807	5 (31.25)	0.802
CG	9 (56.25)		8 (50)		9 (56.25)	
GG	2 (12.5)		1 (6.25)		2 (12.5)	

* Indicates a significant result.

**Table 4:** Allele frequencies for the polymorphisms in study of *NRF2* and *KEAP1*.

	Blood *n* (%)	Surrounding Tissue *n* (%)	*p*-Value OR (95% CI)	Surrounding Tissue *n* (%)	Tumour Tissue *n* (%)	*p*-Value OR (95% CI)	Blood *n* (%)	Tumour Tissue *n* (%)	*p*-Value OR (95% CI) ^1^
*NRF2*									
rs35652124									
A	31 (36.9)	32 (38.1)	0.801 0.881 (0.328–2.367)	32 (38.1)	32 (38.1)	>0.999 1 (0.366–2.730)	31 (36.9)	32 (38.1)	0.801 0.881 (0.328–2.367)
G	11 (13.1)	10 (11.9)		10 (11.9)	10 (11.9)		11 (13.1)	10 (11.9)	
rs6706649									
G	40 (47.6)	31 (36.9)	0.007 * 7.097 (1.465–34.384)	31 (36.9)	36 (42.9)	0.175 0.470 (0.156–1.418)	40 (47.6)	36 (42.9)	0.137 3.333 (0.632–17.574)
A	2 (2.4)	11 (13.1)		11 (13.1)	6 (7.1)		2 (2.4)	6 (7.1)	
rs6721961									
C	38 (45.2)	40 (47.6)	0.397 0.475 (0.082–2.746)	40 (47.6)	39 (46.4)	0.645 1.538 (0.244–9.714)	38 (45.2)	39 (46.4)	0.693 0.731 (0.153–3.485)
A	4 (4.8)	2 (2.4)		2 (2.4)	3 (3.6)		4 (4.8)	3 (3.6)	
*KEAP1*									
rs1048290									
C	19 (29.7)	22 (34.4)	0.434 0.664 (0.238–1.857)	22 (34.4)	19 (29.7)	0.434 1.505 (0.539–4.207)	19 (29.7)	19 (29.7)	>0.999 1 (0.369–2.712)
G	13 (20.3)	10 (15.6)		10 (15.6)	13 (20.3)		13 (20.3)	13 (20.3)	

^1^ OR, odds ratio; CI, confidence interval; * indicates a significant result.

**Table 5:** Genotype frequency Hardy–Weinberg equilibrium for each genotype in different tissues of origin, for the polymorphisms in study of *NRF2* and *KEAP1*.

Type of Tissue	Genotypes Frequency *n* (%)			*p*-Value	
	*NRF2*—rs35652124				
	AA	AG	GG		
Blood	11 (52.40)	9 (42.90)	1 (4.80)	Ref.	
Surrounding Tissue	14 (66.70)	4 (19)	3 (14.30)	0.023 *	Ref.
Tumour Tissue	12 (57.10)	8 (38.10)	1 (4.80)	0.950	0.041 *
	*NRF2*—rs6706649				
	GG	GA	AA		
Blood	19 (90.5)	2 (9.5)	0	Ref.	
Surrounding Tissue	15 (71.4)	1 (4.8)	5 (23.8)	0.055	Ref.
Tumour Tissue	16 (76.2)	4 (19)	1 (4.80)	0.382	0.105
	*NRF2*—rs6721961				
	CC	CA	AA		
Blood	17 (81)	4 (19)	0	Ref.	
Surrounding Tissue	19 (90.5)	2 (9.5)	0	0.378	Ref.
Tumour Tissue	18 (85.7)	3 (14.3)	0	0.679	0.634
	*KEAP1*—rs1048290				
	CC	CG	GG		
Blood	5 (31.3)	9 (56.3)	2 (12.5)	Ref.	
Surrounding Tissue	7 (43.8)	8 (50)	1 (6.3)	0.696	Ref.
Tumour Tissue	5 (31.3)	9 (56.3)	2 (12.5)	>0.999	0.696

* Indicates a significant result.

**Table 6:** Genotype frequency distribution in each one of the tissues and correlation with the breast cancer histological grade.

Type of Tissue	Overall % in Each Histological Grade			*p*-Value	
Blood	*NRF2*—rs35652124				
Histological Grade	AA	AG	GG		
1	3 (42.9)	4 (57.1)	0	Ref.	
2	6 (54.5)	4 (36.4)	1 (9.1)	0.288	Ref.
3	2 (66.7)	1 (33.3)	0	0.390	0.307
Surrounding Tissue					
Histological Grade					
1	3 (42.9)	2 (28.6)	2 (28.6)	Ref.	
2	9 (81.8)	1 (9.1)	1 (9.1)	0.393	Ref.
3	2 (66.7)	1 (9.1)	0	0.188	<0.001 *
Tumour Tissue					
Histological Grade					
1	4 (57.1)	3 (42.9)	0	Ref.	
2	6 (54.5)	4 (36.4)	1 (9.1)	0.809	Ref.
3	2 (66.7)	1 (33.3)	0	0.023 *	0.307
Blood	*NRF2*—rs6706649				
Histological Grade	GG	GA	AA		
1	6 (85.7)	1 (14.3)	0	Ref.	
2	11 (100)	0	0	<0.001 *	Ref.
3	2 (66.7)	1 (33.3)	0	<0.001 *	<0.001 *
Surrounding Tissue					
Histological Grade					
1	5 (71.4)	1 (14.3)	1 (14.3)	Ref.	
2	9 (81.8)	0	2 (18.2)	0.114	Ref.
3	1 (33.3)	0	2 (66.7)	<0.001 *	<0.001 *
Tumour Tissue					
Histological Grade					
1	5 (71.4)	1 (14.3)	1 (14.3)	Ref.	
2	9 (81.8)	2 (18.2)	0	0.002 *	Ref.
3	2 (66.7)	1 (33.3)	0	0.043 *	<0.001 *
Blood	*NRF2*—rs6721961				
Histological Grade	CC	CA	AA		
1	5 (71.4)	2 (28.6)	0	Ref.	
2	10 (90.9)	1 (9.1)	0	<0.001 *	Ref.
3	2 (66.7)	1 (33.3)	0	<0.001 *	<0.001 *
Surrounding Tissue					
Histological Grade					
1	6 (85.7)	1 (14.3)	0	Ref.	
2	10 (90.9)	1 (9.1)	0	<0.001 *	Ref.
3	3 (100)	0	0	<0.001 *	<0.001 *
Tumour Tissue					
Histological Grade					
1	6 (85.7)	1 (14.3)	0	Ref.	
2	10 (90.9)	1 (9.1)	0	<0.001 *	Ref.
3	2 (66.7)	1 (33.3)	0	<0.001 *	<0.001 *
Blood	*KEAP1*—rs1048290				
Histological Grade	CC	CG	GG		
1	1 (16.7)	5 (83.3)	0	Ref.	
2	4 (44.4)	3 (33.3)	22.2	<0.001 *	Ref.
3	0	1 (100)	0	<0.001 *	<0.001 *
Surrounding Tissue					
Histological Grade	CC	CG	GG		
1	1 (16.7)	5 (83.3)	0	Ref.	
2	6 (66.7)	2 (22.2)	1 (11.1)	0.029 *	Ref.
3	0	1 (100)	0	<0.001 *	<0.001 *
Tumour Tissue					
Histological Grade	CC	CG	GG		
1	1 (16.7)	5 (83.3)	0	Ref.	
2	4 (44.4)	3 (33.3)	2 (22.2)	<0.001 *	Ref.
3	0	1 (100)	0	<0.001 *	<0.001 *

* Indicates a significant result.

## Data Availability

The data presented in this study are available within the article or on request from the corresponding author.
